# Transport Property and Spin–Orbit Torque in 2D Rashba Ferromagnetic Electron Gas

**DOI:** 10.3390/ma15155149

**Published:** 2022-07-25

**Authors:** Chao Yang, Da-Kun Zhou, Ya-Ru Wang, Zheng-Chuan Wang

**Affiliations:** 1 College of Mechanical and Electrical Engineering, Wuyi University, Wuyishan 354300, China; 2 School of Physical Sciences, University of Chinese Academy of Sciences, Beijng 100049, China; zhoudakun17@mails.ucas.edu.cn (D.-K.Z.); wangyaru18@mails.ucas.edu.cn (Y.-R.W.)

**Keywords:** 2D Rashba ferromagnetic electron gas, spin-orbit torque, longitudinal conductivity, 72.25.-b, 75.25.-j, 75.76.+j, 85.75.-d

## Abstract

In this paper, we investigate the spin–orbit torque and transport property in a 2D Rashba ferromagnetic electron gas. The longitudinal conductivity can be divided into two parts: the first term is determined by the charge density and is independent of the spin degrees of freedom. The second term depends on the two bands that spin in the opposite directions, and it is directly proportional to spin–orbit torque regardless of the band structure and temperature. This is a general and underlying relation between the transport property and spin–orbit torque. Moreover, we show the impacts of the spin–orbit coupling constant and Fermi energy on transverse conductivity and spin–orbit torque, which is helpful for relevant experiments.

## 1. Introduction

In recent years, spin–orbit torque has become one of the most attractive topics in the field of spintronics because of its great application prospects in magnetic information storage technology [1,2,3]. spin–orbit torque (SOT) is based on spin–orbit interaction (SOI), which uses the non-equilibrium spin accumulation induced by charge flow to generate a torque on a local magnetic moment [4,5], thus achieving the purpose of regulating magnetic storage units. SOT-based magnetic random access memory (SOT-MRAM) overcomes the disadvantages of STT-MRAM, especially the fact that it separates the read and write paths, so it has a higher read and write speed and lower power consumption than STT-MRAM [6,7]. Relevant studies show that SOT-MRAM can achieve ultra-fast information writing, which is reduced from the tens of nanoseconds required by the original STT-MRAM to less than 10 ns, while the power consumption of the device is further reduced [8,9].

The essence of spin–orbit torque is that the directional movement of electrons produces a non-equilibrium spin accumulation in the spin–orbit coupling system, by means of the s–d interaction, the spin accumulation of conducting electrons applies a torque on the local magnetic moment [10,11]. Meanwhile, this directional movement of electrons also generates a charge current in general. Thus, the current and spin–orbit torque are both manifestations of the non-equilibrium transport properties of the SOC system, there must be some relations between them. Literature have showed that the magnitude of spin–orbit torque is often proportional to the current density [12,13,14,15]. Because the spin accumulation and charge current are proportional to the external electric field under linear transport conditions, the ratio depends on the band structure of the system [4,5]. There is no clear conclusion about the underlying relationship between spin–orbit torque and conductivity.

Two-dimensional Rashba ferromagnetic electron gas is an ideal platform for investigating spin–orbit torque [16,17], and the latter is often realized at heavy metal/ferromagnetic interfaces [18,19]. These systems have both Rashba spin–orbit interaction and ferromagnetism, and interesting transport phenomena such as spin–orbit torque and anomalous Hall effect have been found [20,21]. In this paper, we use 2D Rashba ferromagnetic electron gas as an example to explore the relationship between spin–orbit torque and transport properties, as well as the regulation of spin–orbit torque. In Section 2, we show the energy splitting caused by spin–orbital interactions; in Section 3, we study the transport properties of the system, including longitudinal conductance and intrinsic anomalous Hall conductivity; in Section 4, we investigate the spin–orbit torque, and show its regulation in terms of the spin–orbit coupling constant and Fermi energy; Section 5 is the conclusion.

## 2. Model

The Hamiltonian of an electron in a 2D Rashba ferromagnetic electron gas is $\hat{H}=\frac{\hbar^{2}{\vec{k}}^{2}}{2\mu }+\alpha \left(\vec{k}\times \vec{z}\right)\cdot \hat{\vec{\sigma }}-J\vec{M}\cdot \hat{\vec{\sigma }}$, where μ is the effective mass of the electron, $\vec{M}$ is the direction of magnetization and we take an out-of-plane magnetization as: $\vec{M}=\left(0,0,1\right)$, *J* and α are the constant of the s–d interaction and Rashba spin–orbit coupling. To be sure, the s–d interaction is generated between the electron gas (s electrons) and the local magnetic moments (d electrons), where the latter form a three-dimensional ferromagnetic layer [4,5]. Therefore, the Hamiltonian can be written as [16,17,21]:

(1) $$\hat{H}=\left(\begin{array}{cc} \frac{\hbar^{2}k^{2}}{2\mu }-J & \alpha k_{y}+i\alpha k_{x}\\ \alpha k_{y}-i\alpha k_{x} & \frac{\hbar^{2}k^{2}}{2\mu }+J \end{array}\right)$$

Solving the Schrodinger’s equation, we obtain

(2) $$\epsilon_{↑,↓}\left(\vec{k}\right)=\frac{\hbar^{2}k^{2}}{2\mu }\pm \sqrt{J^{2}+\alpha^{2}k^{2}},$$

where $\Delta =\sqrt{J^{2}+\alpha^{2}k^{2}}$ is the energy splitting of these two bands. Correspondingly, the wavefunctions are $\psi_{↑,↓}\left(\vec{k}\right)=e^{i\vec{k}\cdot \vec{r}}{|↑,↓\rangle }_{\vec{k}}$, where

(3) $${|↑,↓\rangle }_{\vec{k}}=\frac{1}{\sqrt{{\left(J\pm \Delta \right)}^{2}+\alpha^{2}k^{2}}}\left(\begin{array}{c} \alpha k_{y}+i\alpha k_{x}\\ J\pm \Delta \end{array}\right).$$

Then, the average spin of the state ${|↑\rangle }_{\vec{k}}$ is $\vec{s}\left(\vec{k}\right){=}_{\vec{k}}\langle ↑|\hat{\vec{\sigma }}{|↑\rangle }_{\vec{k}}=\left(\frac{\alpha k_{y}}{\Delta },\frac{-\alpha k_{x}}{\Delta },\frac{J}{\Delta }\right)$, and the average spin of the state ${|↓\rangle }_{k}$ is ${}_{\vec{k}}\langle ↓|\hat{\vec{\sigma }}{|↓\rangle }_{\vec{k}}=-\vec{s}\left(\vec{k}\right)$.

**Figure 1:**
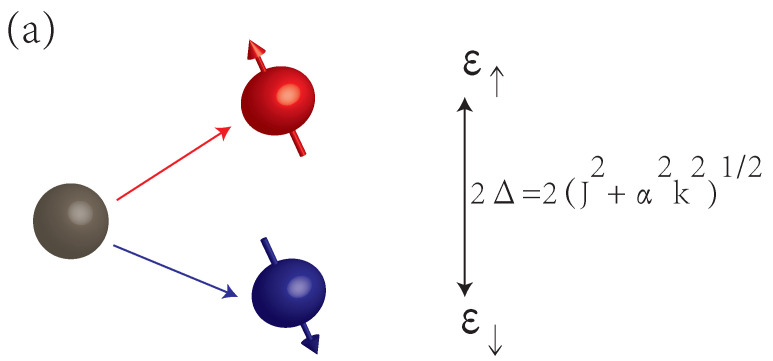

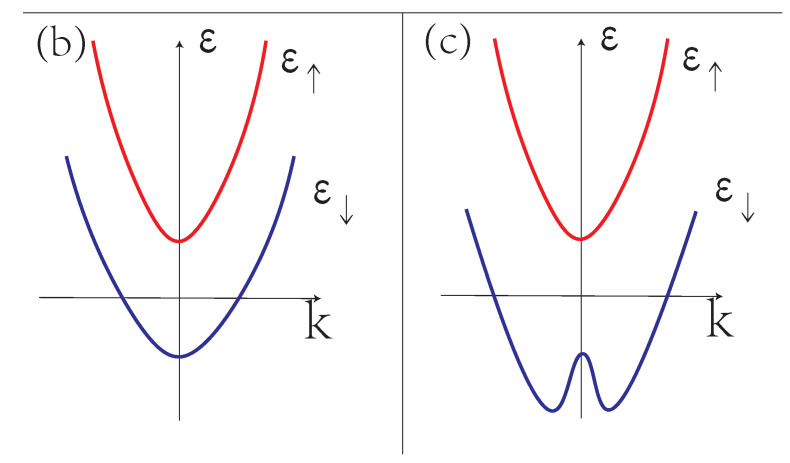
(**a**) Bands splitting with opposite spins; and bands diagram when (**b**) $\alpha k_{J}<J$, (**c**) $\alpha k_{J}>J$.

Figure 1 shows the bands splitting caused by spin–orbit interaction. The spins of electrons in these two bands are in opposite directions, and the energy difference is 2Δ. The band structure depends on the relative magnitudes of α and *J*. If $\alpha k_{J}<J$, where $k_{J}=\sqrt{2\mu J}/\hbar$, $\epsilon_{↑}$ and $\epsilon_{↓}$ are both parabolic. If $\alpha k_{J}>J$, $\epsilon_{↓}$ has a maximum at k=0 and two bottoms. The specific band structures will affect transport property.

## 3. Transport Property

In a semiclassical transport equation, the velocity of electrons in band *n* can be expressed as $v_{n}=\frac{1}{\hbar }\frac{\partial \epsilon_{kn}}{\partial k}-\frac{e\vec{E}}{\hbar }\times {\vec{\Omega }}_{kn}$, where $\frac{1}{\hbar }\frac{\partial \epsilon_{kn}}{\partial k}$ is the traditional velocity and $-\frac{e\vec{E}}{\hbar }\times {\vec{\Omega }}_{kn}$ is the anomalous velocity arising from Berry curvature [22]. In our system, the band splits into $\epsilon_{↑,↓}$, therefore the velocities of electrons are:

(4) $${\vec{v}}_{↑,↓}=\frac{1}{\hbar }{\vec{\nabla }}_{k}\epsilon_{↑,↓}-\frac{e\vec{E}}{\hbar }\times {\vec{\Omega }}_{↑,↓},$$

where $\vec{E}$ is the external electric field and the Berry curvature [22] ${\vec{\Omega }}_{↑,↓}$ can be calculated from wavefunctions: ${\vec{\Omega }}_{↑,↓}\left(\vec{k}\right)=\pm \frac{J\alpha^{2}}{2\Delta^{3}}\vec{z}$.

In a weak external electric field, the distribution functions are $f_{↑,↓}=f_{↑,↓}^{0}+f_{↑,↓}^{1}$, where $f_{↑,↓}^{0}$ are the equilibrium distribution functions, and $f_{↑,↓}^{1}$ is the first-order perturbation caused by an external electric field. By the relaxation time approximation, $f_{↑,↓}^{1}$ can be expressed as: $\frac{e\tau }{\hbar }\vec{E}\cdot {\vec{\nabla }}_{k}f_{↑,↓}^{0}$ and τ is the momentum relaxation time [4]. By the definition of current density $\vec{j}=e\int \left({\vec{v}}_{↑}f_{↑}+{\vec{v}}_{↓}f_{↓}\right)d\vec{k}$, longitudinal and transverse current densities are:

(5) $$j_{x}=\frac{e\hbar }{\mu }\int k_{x}\left(f_{↑}^{1}+f_{↓}^{1}\right)d\vec{k}+\frac{e\alpha^{2}}{\hbar }\int \frac{k_{x}}{\Delta }\left(f_{↑}^{1}-f_{↓}^{1}\right)d\vec{k},$$

and

(6) $$j_{y}=\frac{J\alpha^{2}e^{2}E}{2\hbar }\int \frac{1}{\Delta^{3}}\left(f_{↑}^{0}-f_{↓}^{0}\right)d\vec{k}.$$

To simplify matters, the equilibrium distribution functions can be expressed via the step function at 0K: $f_{↑,↓}^{0}=\theta \left(\epsilon_{F}-\epsilon_{↑,↓}\right)$, where $\epsilon_{F}$ is the Fermi energy. Thus, the longitudinal conductivity is

(7) $$\sigma_{xx}=\frac{e^{2}\tau }{\mu }\int k_{x}^{2}\left(\frac{\partial f_{↑}^{0}}{\partial k_{x}}+\frac{\partial f_{↓}^{0}}{\partial k_{x}}\right)+\frac{e^{2}\alpha^{2}\tau }{\hbar^{2}}\int \frac{k_{x}^{2}}{\Delta }\left(\frac{\partial f_{↑}^{0}}{\partial k_{x}}-\frac{\partial f_{↓}^{0}}{\partial k_{x}}\right),$$

where the first term of Equation (7) can be calculated as:

(8) $$\begin{array}{cc} \sigma_{xx}^{1} & =\frac{e^{2}\tau }{\mu }\int k_{x}^{2}\left(\frac{\partial f_{↑}^{0}}{\partial k_{x}}+\frac{\partial f_{↓}^{0}}{\partial k_{x}}\right)d\vec{k}\\ \; & =\frac{\pi e^{2}\tau }{\mu }\left(k_{F↑}^{2}+k_{F↓}^{2}\right), \end{array}$$

where $k_{F↑,↓}$ is calculated with the formulas: $\frac{\hbar^{2}k^{2}}{2\mu }\pm \sqrt{J^{2}+\alpha^{2}k^{2}}=\epsilon_{F}$. Allowing for the charge density $n=\int \left(f_{↑}^{0}+f_{↓}^{0}\right)=\pi \left(k_{F↑}^{2}+k_{F↓}^{2}\right)$, $\sigma_{xx}^{1}$ can be expressed as $\frac{ne^{2}\tau }{\mu }$ which has the same form with the conductivity of free electron gas. This conductivity is determined by the charge density of electrons, and independent of the spin degree of freedom.

The second term of Equation (7) is derived from the different velocities in two bands:

(9) $$\sigma_{xx}^{2}=\frac{e^{2}\alpha^{2}\tau }{\hbar^{2}}\int \frac{k_{x}^{2}}{\Delta }\left(\frac{\partial f_{↑}^{0}}{\partial k_{x}}-\frac{\partial f_{↓}^{0}}{\partial k_{x}}\right)d\vec{k}.$$

This conductivity results from the different velocities of spin-up and spin-down electrons, and reveals the spin-polarized transport property. Considering the change in Fermi surface, we discuss the results in categories:

When $\epsilon_{F}>J$, the results of equations $\frac{\hbar^{2}k^{2}}{2\mu }\pm \sqrt{J^{2}+\alpha^{2}k^{2}}=\epsilon_{F}$ are $k_{F↑}$ and $k_{F↓}$, respectively. Thus,

(10) $$\sigma_{xx}^{2}=\frac{\pi e^{2}\alpha^{2}\tau }{\hbar^{2}}\left(\frac{k_{F↓}^{2}}{\sqrt{J^{2}+\alpha^{2}k_{F↓}^{2}}}-\frac{k_{F↑}^{2}}{\sqrt{J^{2}+\alpha^{2}k_{F↑}^{2}}}\right).$$

When $-J<\epsilon_{F}<J$, the result of equation $\frac{\hbar^{2}k^{2}}{2\mu }-\sqrt{J^{2}+\alpha^{2}k^{2}}=\epsilon_{F}$ is $k_{F↓}$. Thus,

(11) $$\sigma_{xx}^{2}=\frac{\pi e^{2}\alpha^{2}\tau }{\hbar^{2}}\frac{k_{F↓}^{2}}{\sqrt{J^{2}+\alpha^{2}k_{F↓}^{2}}}.$$

When $\epsilon_{F}<-J$, only if $\alpha k_{J}>J$, the equation $\frac{\hbar^{2}k^{2}}{2\mu }-\sqrt{J^{2}+\alpha^{2}k^{2}}=\epsilon_{F}$ has the solutions $k_{F↓1}$ and $k_{F↓2}$ ($k_{F↓1}>k_{F↓2}$). Thus,

(12) $$\sigma_{xx}^{2}=\frac{\pi e^{2}\alpha^{2}\tau }{\hbar^{2}}\left(\frac{k_{F↓1}^{2}}{\sqrt{J^{2}+\alpha^{2}k_{F↓1}^{2}}}-\frac{k_{F↑2}^{2}}{\sqrt{J^{2}+\alpha^{2}k_{F↑2}^{2}}}\right).$$

**Figure 2:**
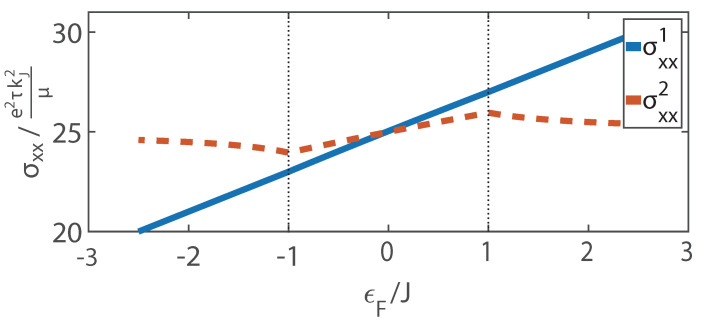
Longitudinal conductivities $\sigma_{xx}^{1}$ and $\sigma_{xx}^{2}$ vs. the Fermi energy $\epsilon_{F}$, where $\alpha k_{J}/J=5$, the solid line is $\sigma_{xx}^{1}$, and the dashed line is $\sigma_{xx}^{2}$.

Figure 2 shows the longitudinal conductivity as a function of Fermi energy $\epsilon_{F}$. With the increase in $\epsilon_{F}$, the charge density *n* increases gradually. Thus, $\sigma_{xx}^{1}$ increases monotonously. At $\epsilon_{F}=\pm 1$, the number of crossover points of the Fermi surface changes. When $\epsilon_{F}=-1$, the Fermi surface intersects with the top of $\epsilon_{↓}$ at k=0. Below this point, there are two Fermi wave vectors $k_{F↓1}$ and $k_{F↓2}$; above that point, $\sigma_{xx}^{2}$ increases with the only Fermi wave vector $k_{F↓}$. Furthermore, when $\epsilon_{F}=1$, the Fermi-surface intersects with the bottom of $\epsilon_{↑}$. Above this point, electrons in band $\epsilon_{↑}$ participate in the conduction process. From Equation (7), we can see a negative contribution made by $\epsilon_{↑}$. Thus, $\sigma_{xx}^{2}$ decreases gradually.

The transverse conductivity is just the intrinsic anomalous Hall conductance, which originates from the Berry curvature [17]. Accordingly, the transverse conductance is

(13) $$\sigma_{xy}=\left\{\begin{array}{cc} \frac{\pi Je^{2}}{2\hbar }\left(\frac{1}{\sqrt{J^{2}+\alpha^{2}k_{F↓}^{2}}}-\frac{1}{\sqrt{J^{2}+\alpha^{2}k_{F↑}^{2}}}\right) & \epsilon_{F}>J\\ \frac{\pi Je^{2}}{2\hbar }\frac{1}{\sqrt{J^{2}+\alpha^{2}k_{F↓}^{2}}} & -J<\epsilon_{F}<J\\ \frac{\pi Je^{2}}{2\hbar }\left(\frac{1}{\sqrt{J^{2}+\alpha^{2}k_{F↓1}^{2}}}-\frac{1}{\sqrt{J^{2}+\alpha^{2}k_{F↑2}^{2}}}\right) & \epsilon_{F}>J \end{array}\right.$$

**Figure 3:**
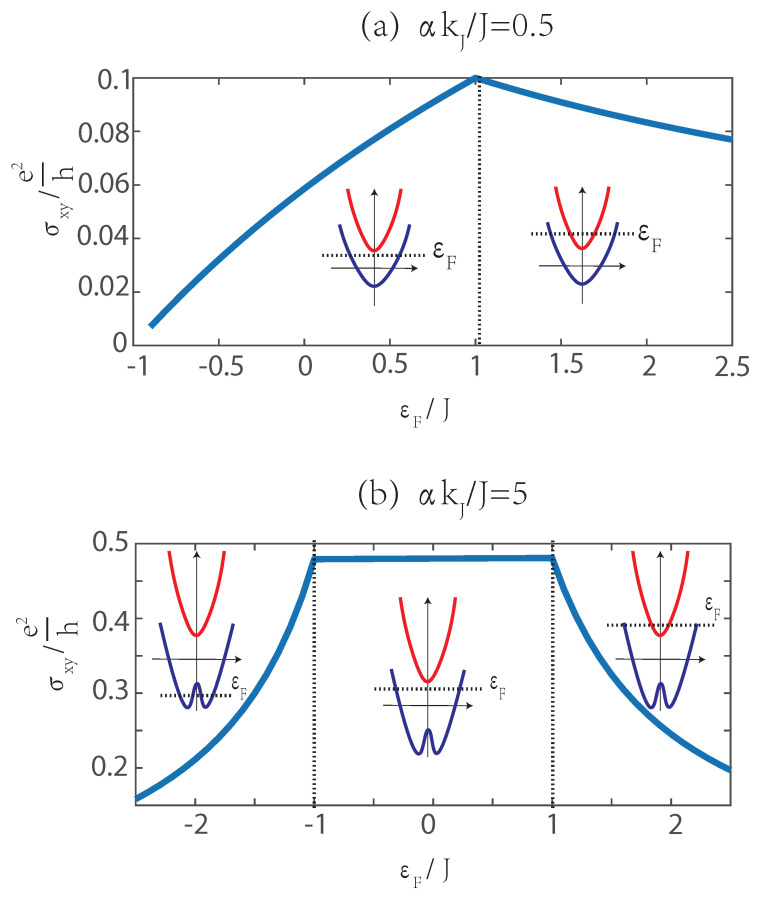
Transverse conductivity $\sigma_{xy}$ vs. the Fermi energy $\epsilon_{F}$, where (**a**) $\alpha k_{J}/J=0.5$ and (**b**) $\alpha k_{J}/J=5$, the red line and blue line are bandstructures of $\epsilon_{↑}$ and $\epsilon_{↓}$ respectively.

Figure 3 shows the transverse conductivity as a function of Fermi energy $\epsilon_{F}$ for different band structures. According to Equation (6), $\sigma_{xy}$ depends on the equilibrium distribution functions $f_{↑,↓}^{0}$, which means that all states below Fermi energy contribute to transverse conductivity. For $\alpha k_{J}<J$, $\epsilon_{↑}$ and $\epsilon_{↓}$ are both parabolic. When $\epsilon_{F}>J$, $\epsilon_{↑}$ begins to make a negative contribution, and thus $\sigma_{xy}$ decreases. For $\alpha k_{J}>J$, $\epsilon_{↓}$ has a top at k=0 and two bottoms. The maximum of Berry curvature is just at k=0, which means a maximum of transverse velocity. Therefore, the transverse conductivity mainly depends on the states around k=0. When $\epsilon_{F}<-J$, $\sigma_{xy}$ increases with the increase in $\epsilon_{F}$, because the states in $\epsilon_{↓}$ around k=0 gradually contribute to $\sigma_{xy}$. When $-J<\epsilon_{F}<J$, $\sigma_{xy}$ is almost unchanged. When $\epsilon_{F}>J$, $\sigma_{xy}$ decreases because of the negative contribution by states in $\epsilon_{↑}$.

## 4. Spin–Orbit Torque

The spin–orbit torque is the result of s–d interaction between the local magnetic moment and spin accumulation of conduction electrons, and it is always expressed as [23,24]:

(14) $$\vec{T}=-\frac{J}{\hbar }\vec{M}\times \vec{m},$$

where $\vec{m}$ is the spin accumulation and can be calculated by definition [4,15]:

(15) $$\vec{m}=\hbar \int \left[\vec{s}\left(\vec{k}\right)f_{↑}-\vec{s}\left(\vec{k}\right)f_{↓}\right]d\vec{k}.$$

According to the distribution function above, the spin accumulation along the y axis is

(16) $$m_{y}=eE\tau \alpha \int \frac{k_{x}^{2}}{\Delta }\left(\frac{\partial f_{↑}^{0}}{\partial k_{x}}-\frac{\partial f_{↓}^{0}}{\partial k_{x}}\right)d\vec{k}.$$

Compared to Equation (9), $m_{y}$ is directly proportional to $\sigma_{xx}^{2}$. $m_{y}/\sigma_{xx}^{2}=\hbar^{2}E/e\alpha$, and this specific value is a constant which has no connection with the band structure and temperature. Thus, the spin accumulation $m_{y}$ and $\sigma_{xx}^{2}$ have the same variation trend in terms of $\epsilon_{F}$.

**Figure 4:**
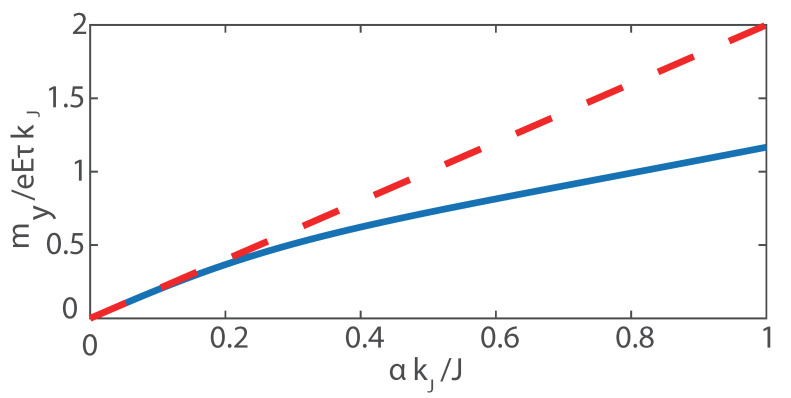
Spin accumulation along the y axis $m_{y}$ vs. Rashba SOC constant α, where the solid line is the result based on the split bands and the dashed line is based on the band of ferromagnetic electron gas.

Figure 4 shows the spin accumulation $m_{y}$ as a function of the Rashba SOC constant α. When α is small, the band structure is still parabolic. Therefore, researchers usually treat the spin–orbit interaction as a perturbation [4,5,25]. The interaction between conduction electrons and the local magnetic moment can be regarded as a spin–orbit effective field acting on local magnetic moment [17,26]: ${\vec{H}}_{R}=\alpha \langle \vec{k}\rangle \times \vec{z}$, where $\langle \vec{k}\rangle$ is the drift of $\vec{k}$ in an external electric field. $\langle \vec{k}\rangle$ is calculated based on the ferromagnetic electron gas model regardless of the band splitting caused by spin–orbit interaction. Consequently, the spin–orbit torque is a direct proportional function of α. When α is large, $\langle \vec{k}\rangle$ is smaller than the result based on the ferromagnetic electron gas model. Our numerical results show the deviation from the proportional relation.

Similarly, the spin accumulation along the z axis is

(17) $$m_{z}=\hbar \int \left[s_{z}\left(\vec{k}\right)\left(f_{↑}^{0}-f_{↓}^{0}\right)\right]d\vec{k}.$$

This spin accumulation depends on the equilibrium distribution function and is parallel to the local magnetic moment; thus, it makes no contribution to the spin–orbit torque.

## 5. Conclusions

In this paper, we investigated the spin–orbit torque and transport property in a 2D Rashba ferromagnet. The main conclusions are as follows:

The longitudinal conductivity can be divided into two parts: the first term is determined by the charge density and is independent of the spin degrees of freedom. The second term depends on the two bands that spin in opposite directions, and this reflects the spin-polarized transport property of the system.

The spin–orbit torque is directly proportional to the second term of longitudinal conductivity, because spin–orbit torque is just caused by the spin-polarized transport. Although this conclusion is obtained at 0K and using parabolic bands, it is suitable for general linear transport cases. This proportionate relation reveals the underlying connection between spin–orbit torque and conductivity.

Moreover, we demonstrate the impacts of the spin–orbit coupling constant and Fermi energy on spin–orbit torque. When these constants are experimentally changed, the spin–orbit torque can be adjusted accordingly. The results are helpful for relevant experiments.

## Data Availability

The data that support the results of this research are available from the corresponding author, [C.Y], upon reasonable request.
